# A novel approach to determine generalist nematophagous microbes reveals *Mortierella globalpina* as a new biocontrol agent against *Meloidogyne* spp. nematodes

**DOI:** 10.1038/s41598-019-44010-y

**Published:** 2019-05-17

**Authors:** Michael J. DiLegge, Daniel K. Manter, Jorge M. Vivanco

**Affiliations:** 1 Center for Rhizosphere Biology, Department of Horticulture and Landscape Architecture, Colorado State University, Fort Collins, Colorado, CO 80523 USA; 2 USDA-ARS, Soil Management and Sugar Beet Research, Fort Collins, Colorado, CO USA

**Keywords:** Fungal pathogenesis, Plant sciences, Agroecology

## Abstract

Root-knot nematodes (RKN) such as *Meloidogyne* spp. are among the most detrimental pests in agriculture affecting several crops. New methodologies to manage RKN are needed such as efficient discovery of nematophagous microbes. In this study, we developed an *in vitro* high-throughput method relying on the free-living nematode *Caenorhabditis elegans* and the infection of those nematodes with a soil slurry containing a microbiome likely to house nematophagous microbes. Nematodes were monitored for presence of infection and sub-cultured repeatedly for the purpose of isolating pure cultures of the microbe responsible for conferring the nematicidal activity. Once soil microbes were confirmed to be antagonistic to *C*. *elegans*, they were tested for pathogenicity against *Meloidogyne chitwoodi*. Using this methodology, the fungal isolate *Mortierella globalpina* was confirmed to be pathogenic *in vitro* against *M*. *chitwoodi* by nematode trapping via hyphal adhesion to the cuticle layer, penetration of the cuticle layer, and subsequently digestion of its cellular contents. *M*. *globalpina* was also observed to reduce disease symptomology of RKNs *in vivo* via significant reduction of root-galls on tomato (*Solanum lycopersicum* var. Rutgers).

## Introduction

Members of the phylum Nematoda have been in existence for close to one billion years and are classified as one of the most diverse organisms globally^1^. Soil-borne nematodes feed on nearly all forms of life, including bacteria, fungi, unicellular eukaryotes, plants, and animals^2^. Most herbivorous or plant-parasitic nematodes (PPNs) affect crops through feeding within or on plant roots^3^. However, PPNs of economic importance can be grouped by either causing direct damage to their host or acting as a vector for viruses and/or bacteria^3^. According to Kaplan, nematodes are annually responsible for ~$100 billion in crop damage and cause 5 percent of all yearly crop loss globally. *Meloidogyne* spp., or the root-knot nematodes (RKN), are able to infect a wide range of crop families such as Solanaceae, Brassicaceae, Leguminosae, Musaceae, Curcubitaceae, Poaceae, and others^4,5^. Within their several plant hosts, RKNs possess the ability to dedifferentiate plant cells into multinucleate feeding sites via hypertrophy by synthesizing specific effectors within their esophageal gland. These effectors are then injected into the host plant cell – altering the cell’s DNA to establish a swollen feeding cell^5^.

Attempts to control nematode pests have been practiced by means of soil sterilization (via fumigation or solarization), crop rotation, and resistance breeding^6,7^. These control practices have become increasingly difficult over the years. For fumigation, the U.S. Environmental Protection Agency phased out the use of the chemical nematicide methyl bromide in 2005^8^. Introgression of nematode resistance genes is common in rootstock production for many woody plants but can be an impractical or limited process with annual crops due to the few available RKN-resistance genes, and lack of ability to deploy these into the many susceptible crops^4,9,10^. Crop rotations have served growers little success as RKN members have a wide variety of plant hosts^4,11^. In organic systems, soil amendments along with solarization practices have been attempted to control RKN, yielding the most success when used simultaneously^12^. Unfortunately, within these studies - nematode populations near the margins of the solarized beds were observed to recover during the growing season^12^.

In efforts to combat these difficulties, a limited number of biological products have been formulated that tend to contain *Pasteuria nishizawae* or *Purpureocillium lilacinum* strain 251^13,14^. However, *P*. *nishizawae* has only been observed to complete its life cycle in the adults of the soybean cyst nematode. Although *P*. *lilacinum* strain 251 is confirmed to have no human risks, other species of *P*. *lilacinum* are known to produce mycotoxins and paecilotoxins which are harmful to humans^15,16^. It should be noted that the reproducibility of nematode control sought after by these commercial microorganisms in agronomic systems appears to be lacking^17^. Thus, there is a need to isolate new and more efficient microbes that are antagonistic against RKNs.

Plant-parasitic nematodes require a plant host to complete their reproductive cycle, and as such, the establishment of *in vitro* RKN cultures is a lengthy multi-step process^18^. The method of culturing RKNs *in vitro* makes very difficult the development of a high-throughput system to screen for new microbes that could exhibit root knot nematicidal properties. Unlike common PPNs, *Caenorhabditis elegans* is a model bacterivorous nematode easily grown *in vitro*^19^. A series of methodologies have been designed to utilize *C*. *elegans* as a model for screening virulence genes of certain human pathogens such as *Pseudomonas aeruginosa* and several others^20–23^. In the present study, it was hypothesized that nematophagous microorganisms would not discriminate against different species of nematodes that practice different feeding habits as the cuticle layers of both bacterivores (*Caenorhabditis* spp.*)* and RKNs (*Meloidogyne* spp.*)* are largely composed of collagen^24^. In support of our hypothesis, chemical nematicides such fluensulfone have been found to induce negative effects on both *Caenorhabditis* and RKNs^25^. To test this hypothesis, we used a soil containing microbes likely to have a negative effect against PPNs^26^.

Here, we developed a novel method to circumvent the use of PPNs under *in vitro* conditions in order to develop a high-throughput screening method of generalist pathogens against nematodes. Our methodology consisted of creating a soil slurry of microbes and applying it to petri dishes containing the easily-culturable *C*. *elegans*. The screening of microbial candidates on *C*. *elegans* as opposed to RKNs expedited the process of antagonistic microbe identification. With this method, we were able to rapidly isolate an antagonistic fungus of *C*. *elegans*. Subsequently, the fungus was tested and confirmed to be antagonistic against the highly aggressive RKN of potatoes, *Meloidogyne chitwoodi* both *in vitro* and *in vivo*.

## Results

### Screening of microbes using *C. elegans* methodology

A total of 16 microbial isolates were recovered from the process of transferring paralyzed and/or dead nematodes to sterile media, post-inoculation of slurries from 3 different soil sources. For more information on the preliminary screening process, see Supplementary Note S1 and Fig. S1. Microbes growing around dead nematodes were determined to be bacterial or fungal based on visual appearances. For instance, if there were biofilm growing around the paralyzed nematode - the nematode was transferred to Luria-Bertani Agar (LBA) as it was assumed to be of bacterial origin. In contrast, if hyphae were actively growing around the paralyzed nematode, the worms were removed from the culture and placed into Potato Dextrose Agar (PDA) to recover the fungus. A challenge brought upon by using this methodology was determining if the nematode’s death was caused by one single microorganism or a combination of microbial action. This issue was overcome by isolating the 16 initial microbial candidates three times in order to obtain pure cultures of the candidate nematophage(s). Organisms were grown at 26 °C for at least 24 hours and once pure broth cultures of all isolates were obtained, the microbial isolates were inoculated onto high population *C*. *elegans* plates and monitored for nematode parasitism/antagonism. The inoculation process of pure culture was repeated 3 times for each microbe in order to confirm the successful nematode parasitism/antagonism brought upon by the microbial candidates. After testing all of the isolates independently of one another, only the most effective fungal candidate was identified and investigated further for its nematicidal properties against RKNs (Supplementary Table S1). The fungus was identified as *Mortierella globalpina*.

*Mortierella globalpina* expressed the most rapid and effective nematode pathogenesis compared to the other microbes tested in this study. The other fifteen isolates expressed no effect or limited pathogenesis on *C*. *elegans* when tested in isolation as compared to the visible immobilization and subsequent nematophage behavior brought upon by *M*. *globalpina* (see Supplementary Data for additional information).

### *In vitro* experimentation of *Mortierella globalpina* on *Caenorhabditis elegans*

It was determined that *C*. *elegans* populations were effectively decimated with the inoculation of *M*. *globalpina* spores to the nematode culture. After fungal inoculation, both adult and juvenile nematodes, as well as eggs of *C*. *elegans*, were monitored over the course of 72 hours at room temperature (28.8 °C ± 2 °C) until the fungus had completely colonized the petri dish leaving only paralyzed nematodes. It was observed that the fungus fully infected all life stages of *C*. *elegans in vitro* in up to 72 hours (Supplementary Table S1). In other words, *M*. *globalpina* infected eggs, larval-stages (Larval stage 1 [L1] to L4) and adults (nematodes capable of egg laying) of *C*. *elegans*.

### Identification of nematophagous fungus

The genetic sequence of the fungal isolate in question was confirmed to be *Mortierella globalpina* via a matching of the 479 base pairs to a sequence in the public database GeneBank with 100% similarity (NCBI Accession No. NG_064079).

### Experimentation of *Mortierella globalpina* with *Meloidogyne chitwoodi*

The nematophagous nature of *M*. *globalpina* observed against *C*. *elegans* was once again observed *in vitro* during pathogenicity experimentation with *M*. *chitwoodi* (Supplementary Table S1). In just 72 hours of exposure to the fungus, the populations of *M*. *chitwoodi* nematodes were immobilized by *M*. *globalpina*. In two of the five replicate-populations (containing approximately 30 ± 14 nematodes), all nematodes were immobilized. In the remaining three replicates, only two nematodes per replicate survived parasitism by *M*. *globalpina* within the first 72 hours (Supplementary Table S1). Over the course of 72 hours it was observed that *M*. *globalpina* was pathogenic to both the eggs and juvenile stages (Juvenile stage 2 [J2] – J4), as well as adult nematodes (capable of reproduction) of *M*. *chitwoodi in vitro*.

### Scanning electron microscope imaging

**Figure 1:**
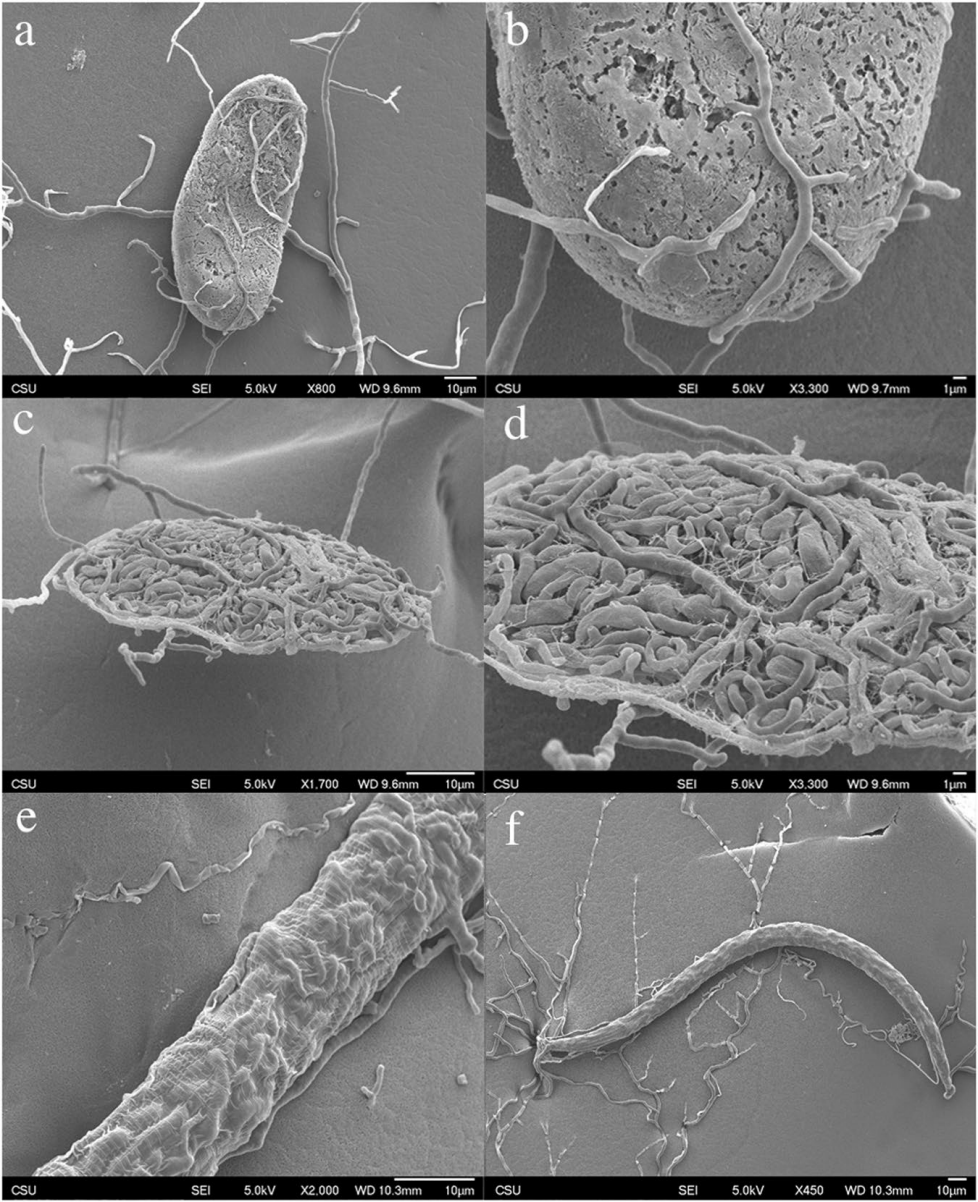
Scanning electron micrographs of *Mortierella globalpina* pathogenesis on *Meloidogyne chitwoodi* nematodes. (**a**) Hyphae of *M*. *globalpina* penetrating egg of *M*. *chitwoodi*. (**b**) Higher magnification of hyphae from *M*. *globalpina* growing and penetrating egg of *M*. *chitwoodi* (**c**) Hyphae from *M*. *globalpina* growing inside *M*. *chitwoodi* egg and digesting cuticle layer of *M*. *chitwoodi* egg. (**d**) Higher magnification of hyphae from *M*. *globalpina* growing inside *M*. *chitwoodi* egg and digesting inner contents of egg. (**e**) *M*. *globalpina* hyphae fully colonizing inner-cuticle of *M*. *chitwoodi* adult and presumptively digesting cellular contents. (**f**) Hyphae of *M*. *globalpina* colonizing inner contents of adult *M*. *chitwoodi* nematode to then reemerge from nematode cuticle to continue search for nutrition. Six images were combined into one figure with Adobe Photoshop CS6.

Under the electron microscope, the fungus was confirmed to infect both eggs, larvae, and adults of *M*. *chitwoodi* (Fig. 1). It was observed that the fungal mycelium attached and surrounded the eggs of *M*. *chitwoodi*. Similar types of attacks were observed on larvae and adults (Fig. 1). In all instances, the mycelium was able to penetrate the cuticle and presumably digest the cellular contents of the nematodes and/or the eggs.

### The effect of *M. globalpina* on rutgers var. tomato plant growth parameters

**Figure 2:**
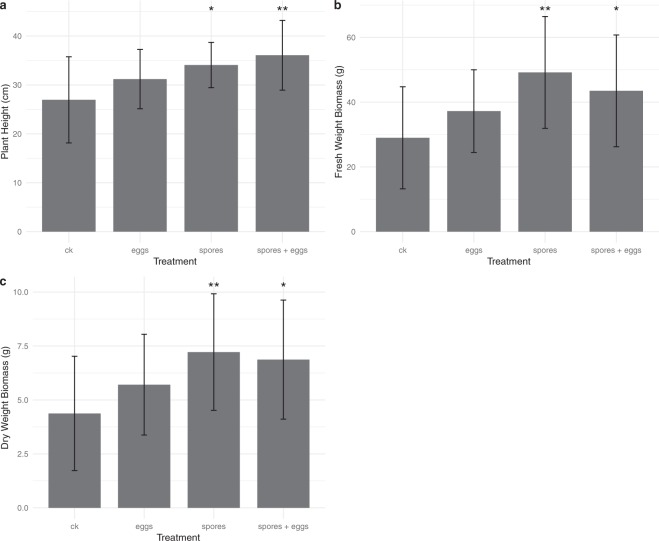
Total height and biomass (fresh and dry) of Rutgers var. tomato plants on harvest day (average of 15 replicates, displayed as mean ± standard deviation). (**a**) Height. *M*. *globalpina* spores and *M*. *globalpina* spores plus *M*. *chitwoodi* eggs treatments both showed significant height increases compared to the control (+7.13 cm and +9.12 cm, respectively) (p-values: 0.03, and 0.003 respectively, Tukey test). (**b**) Total fresh-weight biomass of treated Rutgers var. tomato plants on harvest day. *M*. *globalpina* spores-inoculated treatment showed significant fresh-weight increases compared to the control (+20.20 g) (p-value: 0.005, Tukey test). (**c**) Total dry-weight biomass of Rutgers var. tomato plants on harvest day. *M*. *globalpina* spores treatment showed significant dry-weight increases compared to the control (+2.83 g) (p-value: 0.02, Tukey test).

The total plant height as well as fresh and dry weight biomass of tomato was recorded upon harvest. The plants treated with only *M*. *globalpina* spores as well as the *M*. *globalpina* spores plus *M*. *chitwoodi* eggs group expressed significant height increases compared to the control (no applications of *M*. *globalpina* nor *M*. *chitwoodi*) (p-values: 0.030, 0.003 respectively) (Fig. 2a). Additionally, the treatment inoculated with spores of *M*. *globalpina* showed significant fresh-weight increases compared to the control (p-value: 0.005) (Fig. 2b). The *M*. *globalpina* spores treatment also expressed a significant increase in dry-weight biomass production compared to the control (p-value: 0.022) (Fig. 2c).

### Root scanning analysis

**Figure 3:**
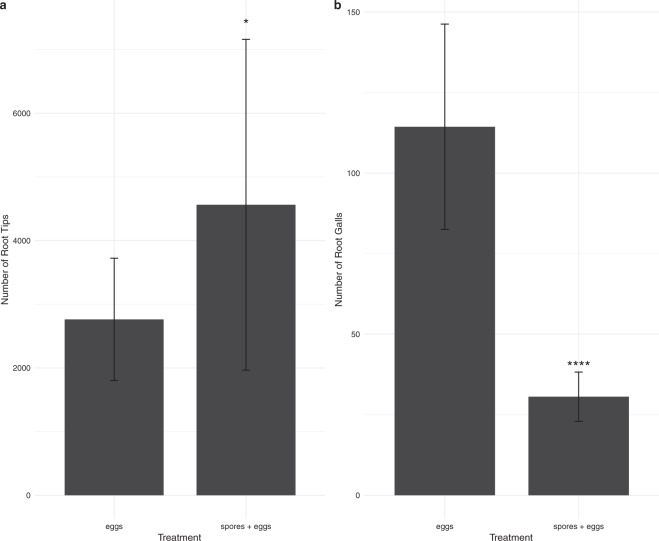
WinRHIZO Root scan analysis (average of 15 replicates, displayed as mean ± standard deviation): (**a**) Root Tips. *M*. *globalpina* spores plus *M*. *chitwoodi* eggs*-*inoculated plants significantly increased number of root-tips compared to *M*. *chitwoodi* eggs-only inoculated treatment (+1800.4 root tips) (p-value = 0.018, Tukey test). (**b**) Root Galls. Root scan analysis of number of root galls (gall = PPN infection site). *M*. *globalpina* spores plus *M*. *chitwoodi* eggs*-*inoculated treatment significantly decreased the number of root galls compared *M*. *chitwoodi* eggs-only inoculated treatment (−83.8 galls) (p-value: 1.19e^−10^, Tukey test).

Only the roots of the *M*. *chitwoodi* egg-inoculated plants and those of the *M*. *globalpina* spores plus *M*. *chitwoodi* eggs inoculated treatments were subject to root scanning and analysis. This is because all plants subject to nematode infection could be analyzed for root growth abnormalities, whilst the *M*. *globalpina* spore-only inoculated plants were used to monitor for any plant growth promoting fungal (PGPF) effects on tomato. Of all parameters analyzed (see Supplementary Table S2), plants inoculated with *M*. *globalpina* spores plus *M*. *chitwoodi* eggs performed significantly better than *M*. *chitwoodi* eggs only treatment. The spores plus eggs treatment resulted in increased number of root-tips (Fig. 3a) and reduced number of root-galls (Fig. 3b) compared to the *M*. *chitwoodi* eggs only inoculated treatment (p-values: 0.0178, and 1.19e^−10^ respectively). In this study, the reduced number of nematode infection sites (galls) within tomato roots was an indicator of effective biocontrol of RKNs.

## Discussion

In recent years, chemicals like 1,3-dichloropropene (1,3-D) and metam sodium have been utilized as alternatives to the use of methyl bromide^27^. It was found that the most promising chemical alternatives to methyl bromide for PPN control were the combinations of 1,3-D-chloropicrin, chloropicrin-proprietary solvent, and 1,3-D-metam sodium^27^. However, compounds like metam sodium can have adverse ecological and human health effects^28^. Pruett *et al*. suggests that metam sodium can induce both allergic dermatitis and/or asthma in humans^28^. Thus, ecologically friendly alternative methods to control PPN (such as beneficial microbes) are needed.

There is palpable need to identify new microbes that could control plant parasitic nematodes of economic importance^29^. However, certain difficulties arise in the search for new nematophagous microorganisms such as the lack of a widely used pathogenicity-screening method. In this study one of our aims was to develop a simple and high-throughput methodology to identify new beneficial microbes that could serve as general-antagonists against nematodes. Although ideally any screening would be target specific, the difficulty in culturing PPNs *in vitro* makes this unreasonable. This difficulty has also resulted in chemical nematicides that are non-specific. However, by using a free-living nematode model, we reasoned that we could develop a targeted approach that is limited to nematodes. Both PPNs and free-living nematodes share the same soil habitat and are subject to similar pressures such as the presence of pathogens. Accordingly, it has been shown that different types of soil nematodes were affected similarly when mammalian grazing was introduced to the above-ground land of their soil habitat^30^. Similarly, nematodes of different orders experienced analogous population decline or elimination as a result of increasing heavy metal contents in an agroecosystem soil^31^. These studies contribute to the assumption that nematodes of different genera are susceptible to similar stresses.

We used the soil microbiome as a natural reservoir of potential microbial biocontrol agents against PPN. We specifically selected a soil (Sargent farm) that has been previously analyzed in terms of microbiology and showed low ratios of PPN^26^. The total nematode faunal profile in the Sargent farm soil proposed by Castillo *et al*. was 10% Herbivores, 62% bacterivores and 28% fungivores. Based on this nematode profile, the soils of the Sargent farm fit into ‘Quadrat D’ of the soil food web proposed by Ferris *et al*.^26,32^. Characteristics of the ‘Quadrat D’ show a food web that is generally degraded and includes both a high C:N ratio and fungi being the primary driver of decomposition^32^. With the Sargent farm soil expressing a low population of herbivorous nematodes as well as fungi being the primary source of decomposition, we hypothesized that this soil was likely to contain a fungal community pathogenic to PPNs.

*Caenorhabditis elegans* was used as a model organism in our study because it is easily established and commonly cultured under *in vitro* conditions^33^. In further support of the use of *C*. *elegans* as a model organism, the nematode has also been used as a model in other studies such as high-throughput drug discovery for anti-microbials, anti-infectives, immunity responses, host-pathogen interactions, and several other bioactive responses^34–37^. Interestingly, Hsueh *et al*. utilized *C*. *elegans* to characterize the attraction and trapping ability of the fungus *Arthrobotrys oligospora*^38^. However, the fungus *A*. *oligospora* had been characterized previously by Fresenius in 1850 and *C*. *elegans* was merely used to visualize and photograph the nematophage in action^38,39^.

In our study, the fungus *M*. *globalpina* was selected as the candidate for experimentation as it was observed to parasitize eggs, larvae and adults of *C*. *elegans* nematodes. Additionally, *M*. *globalpina* also expressed effective nematode pathogenicity in its isolated form rather than in combination with other microbes. There is little information about the members of the fungal genus *Mortierella* serving as nematophagous fungi. Orion *et al*. reported that *Mortierella* spp. was observed inside the gelatinous matrix of RKN egg masses^40^. *Mortierella* spp. hyphae were also observed in hatched eggs of RKN, suggesting a nematophagous nature by one or several fungi of the *Mortierella* genus^40^. However, the member *Mortierella alpina* was tested in greenhouse studies against *M*. *javanica*, but was most effective when co-inoculated with *Trichoderma longibrachiatum*^41^.

Our results suggest that *M*. *globalpina* produces extracellular enzymes that can degrade the cuticle of both *C*. *elegans* and *M*. *chitwoodi*. It is worth noting the cuticle of most nematodes is largely composed of collagen^24^; thus, it is likely that *M*. *globalpina* might produce and secrete collagenases. This report is to the best of our knowledge the first time that *M*. *globalpina* has been introduced as a biocontrol agent of RKNs. Additionally, the application of *M*. *globalpina* spores alone increased root and shoot biomass of Rutgers var. tomato plants compared to other treatments. This suggests that *M*. *globalpina* may have some plant growth promoting effects on tomato. Although not tested here, *Mortierella antarctica* and *Mortierella verticillata* have been observed to synthesize gibberellic acid and indoleacetic acid *in vitro*, which may explain the growth promoting effects on tomato of *M*. *globalpina*^42^.

In conclusion, this study reports a new method to rapidly identify generalist nematode pathogens by utilizing the free-living *C*. *elegans* as a model. In addition, we state that a newly-isolated nematophagous *Mortierella* globalpina reduced nematode infection sites (galls) and could potentially serve as a useful biocontrol agent against RKNs in cropping systems. However, further field studies are necessary to determine (i) the usefulness of *M*. *globalpina* as a biocontrol measure on other herbivorous nematodes (non-RKNs), (ii) the ecological impacts on the natural soil microbiota when using *M*. *globalpina* as a biocontrol agent, (iii) the effect of *M*. *globalpina* inoculation as a protective measure in known PPN/RKN-infested soils, and (iv) the mechanism of chemical action for the pathogenesis of *M*. *globalpina* against *M*. *chitwoodi*.

## Methods

### Experimentation with *Caenorhabditis elegans*

#### Soil slurry preparation and inoculation

In screening for nematophagous microbes we used the ‘Sargent’ potato field soil listed in Castillo *et al*., which has a strong likelihood of containing beneficial microbes^26^. A two-part ‘Sargent’ soil and one-part molecular water slurry was prepared. This slurry functioned as a liquid suspension of microorganisms present within the Sargent field soil. The water and soil mixtures were contained in a 50 ml VWR Falcon tube and were homogenized for 2 hours on a Fisher Vortex Genie at maximum speed. The slurry was then centrifuged at 2440 RPM in a Sorvall Super T21 benchtop centrifuge for 10 minutes at room temperature (28.8 °C ± 2 °C) to pellet any remaining soil debris. Subsequently, the slurry was aspirated from the Falcon tube, and serially diluted to prepare 10^−6^, 10^−7^ and 10^−8^ dilutions. Aliquots of 50 ul and 100 ul of each slurry dilution were plated onto high population (>1,000) *C*. *elegans* culture plates (described in 4.1.2) to monitor for microbial pathogenesis (Supplementary Fig. S1). The studies were repeated three times using three replicates for each solution/dilution in order to locate as many nematophagous microbial candidates as possible. For a full description of this methodology and soils tested in preliminary experimentation, see Supplementary Note S1.

#### Population establishment of *Caenorhabditis elegans*

*Caenorhabditis elegans* nematodes were obtained from the Biochemistry Department at Colorado State University in Fort Collins, CO. Nematodes were cultured *in vitro* on 9 cm petri dishes prepared with Nematode Growth Media (NGM) agar^33^. Nematode growth media agar consists of: 3 g NaCl, 2.5 g bacterial peptone, 17 g agar and 975 ml of diH2O. After autoclaving, the following compounds are mixed into the NGM: 1 ml of 1 M CaCl_2_, 1 ml of 1 M MgSO_4_, 25 ml of 1 M KPO_4_ and 1 ml cholesterol in ethanol (5 mg/ml). To provide a food source for *C*. *elegans*, *Escherichia coli* OP50 was grown onto Luria Bertani agar (LBA) at 37 °C until a bacterial lawn was established. Once the lawn was formed, an 8 mm by 8 mm square chunk of *E*. *coli* OP50 was excised from the LBA and seeded onto the NGM agar. The seeded NGM was incubated at 37 °C until the *E*. *coli* formed a new bacterial lawn. After bacterial establishment, the petri dishes were inoculated with 30 ul suspension of *C*. *elegans* (containing 10 ± 4 eggs or stage 1 larvae (L1) of bacterivorous nematodes) obtained using a common nematode synchronization methodology^33^.

#### Determining microbial pathogenicity against *Caenorhabditis elegans*

*Caenorhabditis elegans* nematodes assumed to be antagonized by nematophagous microorganisms were examined for behavioral difficulties such as lack of movement and/or nematode body stiffening. Those nematodes expressing damaged cuticle layers, and/or presence of bacteria [biofilm] or fungi [hyphae] around the body were identified. The nematodes that were infected were removed from the NGM via the use of a needle from an *Echinopsis pachanoi* cactus mounted with parafilm to a wooden stirrer. Once located, the affected nematodes were placed onto either Potato Dextrose Agar (PDA) for fungal isolates or Luria Bertani Agar (LBA) for bacterial isolates to subsequently isolate individual colonies. Isolates were repeatedly sub-cultured, at least 3 times, until a pure culture was obtained and used for further experimentation. Bacterial isolates were incubated in the dark at 37 °C and fungal isolates were incubated in the dark at room temperature (28.8 °C ± 2 °C).

#### Confirmation of microbial-pathogenicity against *C. elegans*

After obtaining pure cultures from the isolates assumed to be antagonistic or pathogenic to *C*. *elegans*, the microbes were cultured into a broth (liquid media, PD broth or LB broth). The liquid isolates were grown to a concentration of 1 × 10^8^ spores or cells/ml and then inoculated onto *C*. *elegans* plates. Inoculated plates were examined over the course of 72 hours in order to monitor and confirm any antagonism or pathogenic attacks against *C*. *elegans in vitro*. Microbial pathogenicity confirmation was repeated in triplicate prior to testing the isolated organism(s) against *Melodogyne chitwoodi*.

### Experimentation with *Meloidogyne chitwoodi*

#### *In Vitro* experimentation

Rutgers var. tomato seeds were surface sterilized with a 2.5% sodium hypochlorite (NaClO) solution, rinsed 5 times with sterile water and sown into water-agar (10 grams per liter, pH 7.4) at four seeds per petri dish (See Supplementary Image S1). After one week submerged in the agar, when the length of the roots ranged from 1 cm-2 cm long, *in vitro* experimentation began. Fungal spores were cultured in liquid (Potato dextrose broth) and were inoculated onto the agar surrounding tomato roots at 20 uL/root at a concentration of 1 × 10^8^ spores/ml. Additionally, 30 ± 14 *M*. *chitwoodi* eggs were then added in the center of the 9 mm petri dish to examine effects of the fungal isolate as a biocontrol agent against *M*. *chitwoodi* nematodes. Five replicates of this experiment were prepared for testing, and the experiments were repeated in triplicate to confirm results. Petri dishes were monitored for 72 hours to examine signs of plant parasitism by nematode, or nematode pathogenesis by *M*. *globalpina*. Observations of infected or immobilized nematodes were recorded until all present nematodes were infected by the fungus (~72 h).

#### Identification of nematophagous fungal isolate

A pure culture of a nematophagous fungus with pathogenic activity against both *C*. *elegans* and *M*. *chitwoodi* was cultured on sterile PDA and grown for 24 hours in the dark at room temperature (28.8 °C ± 2 °C). Freshly grown samples were then sent to MIDI labs (Newark, DE) where the 28s large subunit (LSU) rRNA gene sequencing was conducted in order to obtain the organism’s identity. The protocol to generate the 28s rRNA gene sequence is as follows: Approximately 300 base pairs of the variable D2 region of the LSU rRNA gene is PCR amplified from genomic fungal DNA. The primers used for the PCR and sequencing correspond to positions 3334 F and 3630 R in the *Schizosaccharomyces japonicus* LSU rRNA gene. Amplification products are purified from excess primers and dNTPs and checked for quality and quantity by running a portion of the products on an agarose gel. Cycle sequencing of the 16S rRNA amplification products were carried out using DNA polymerase and dye terminator chemistry. Excess dye-labeled terminators were then removed from the sequencing reactions. The samples were electrophoresed on either a 3,130 or 3,130xl Genetic Analyzer

#### *In Vivo* experimentation

Rutgers var. Tomato seedlings were pre-germinated in Promix Bx peat moss and grown in plug trays until two sets of true leaves had emerged on the seedlings. This experiment consisted of three treated groups of tomato plants and a control, and all groups consisted of 15 replicates. Treatments were determined in order to observe the effect of *M*. *globalpina* on tomato plants infected with PPN. The treatments were: *M*. *chitwoodi* eggs, *M*. *chitwoodi* eggs plus *M*. *globalpina* spores, and *M*. *globalpina* spores alone, and control group. In all treatments; roots of tomato seedlings were washed of adhering substrate and suspended in either *M*. *globalpina* spores cultivated in PDB at [1 × 10^8^ spores/ml], sterile PDB or sterile water for 20 minutes^41^. After root-dip inoculation, plants were transplanted into a (1:1:1) Promix Bx: Vermiculite: Sand substrate to allow for effective nematode movement throughout. After transplanting tomato seedlings, *M*. *chitwoodi* eggs were added at 10,000 eggs/plant. The eggs were inoculated into the substrate at the soil level near the root zone by the methodology described by AL-Shammari *et al*.^41^. Plants that underwent fungal-root-dip inoculation (but were not infected with eggs of *M*. *chitwoodi*) were carried out in order to monitor for any plant growth promoting fungal effects on tomato. The experiment was conducted in CSU’s Horticulture Center Greenhouse and carried out in a randomized block design provided by random.org, with each plant pot serving as a block. This *in vivo* experiment was repeated 5 times in order to confirm results. Plants were grown for a total of 7 weeks and harvested 5 weeks after the nematode inoculation. Upon harvesting, fresh and dry weight biomass (above-, below-ground, and total) were recorded, along with the final plant height. Additionally, the roots of the plants exposed to *M*. *chitwoodi* eggs were washed of all substrate prior to biomass recording and were scanned and analyzed by WinRHIZO Pro software. After analysis of roots, all plant matter was oven dried at 70 °C for until there was no change in dry weight biomass measurement.

### Root scanning and analysis

Only roots of plants treated with *M*. *chitwoodi* eggs were scanned for analysis. Tomato plants were uprooted on harvest day and roots were washed free of any adhering substrate. Roots were then removed from shoots, submerged into shallow trays of water and scanned using an EPSON Perfection V750 Pro Photo Scanner. Once the images of roots were acquired, they were analyzed by Reagent Instrument Inc.’s WinRHIZO Pro. The total root length, surface area, average root diameter, root volume, number of crossings, the number of root galls, and the number of root forks were observed and analyzed via the use of WinRHIZO Pro analysis software. In our study, number of galls were used as a measure to show effectiveness (or lack thereof) of *M*. *globalpina* in controlling RKNs, and reduced symptomology of nematode infection (or a reduction in the number of root-galls) was an indicator of biocontrol agent effectiveness.

### Electron microscope imaging

The fungus *M*. *globalpina* was grown on 5 cm petri dishes filled with water-agar (pH 7.4) and planted with four Rutgers var. tomato seeds similar to the methodology in 4.2.1, but smaller (5 cm) petri dishes. Once the mycelium began to grow onto the tomato roots; eggs and L1 larvae of *M*. *chitwoodi* were added to the center of the water agar dishes. The petri dishes were sealed with parafilm and placed in the dark at 26 °C. Plates were examined over the course of 26 hours and any fungal attacks on the nematodes or eggs were excised from the agarose plates and stored in a glutaraldehyde fixative (pH 7.4) at 4 °C for later SEM processing. For SEM success, the samples needed to be 1 mm^3^ and were prepared with a No. 10 scalpel blade under a laminar flow hood. The samples were then processed through critical point drying, mounting, and subsequently imaged on a Scanning Electron Microscope at Colorado State University’s Central Instrument Facility. Samples were prepared in 50 mm agar petri dish with fungus, nematodes and newly germinated tomato plant roots.

### Statistical analysis

The differences between groups in all results were analyzed by one-way ANOVA and if significant, were further analyzed with Tukey’s HSD test with R statistical analysis software. Figures showing significance in both plant growth parameter and root scan results were created with R statistical software.

## Supplementary information

Supplementary Information

## Data Availability

The datasets generated during and/or analyzed throughout the current study are available from the corresponding author upon reasonable request.
