# Supplementary material for: A novel approach to determine generalist nematophagous microbes reveals Mortierella globalpina as a new biocontrol agent against Meloidogyne spp. nematodes
Source: Sci Rep. 2019 May 17;9:7521. doi: 10.1038/s41598-019-44010-y (PMC6525257; doi:10.1038/s41598-019-44010-y)
Supplement: Supplementary file 1 — Supplementary Information [file 41598_2019_44010_MOESM1_ESM.pdf]

A novel approach to determine generalist nematophagous microbes reveals *Mortierella globalpina* as a new biocontrol agent against *Meloidogyne* spp. nematodes

Michael J. DiLegge[1], Daniel K. Manter[2], Jorge M. Vivanco[1*]

[1]Center for Rhizosphere Biology, Department of Horticulture and Landscape Architecture, Colorado State University, Fort Collins, CO 80523, USA.
[2]USDA-ARS, Soil Management and Sugar Beet Research, Fort Collins, CO. USA
*Correspondence: Jorge M. Vivanco
Email: j.vivanco@colostate.edu
217 Shepardson Building, 301 University Avenue, Fort Collins, CO 80523
Telephone: +1 (970)491-7170

Supplementary Note S1: Description of preliminary microbial screening from three distinct potato field soils.

We began this experiment by using three different potato-field soils with variable counts of herbivorous nematodes to create the initial soil slurry inocula (Mosca II at 9% PPN of total community, Sargent at 10% and Blanca at 6%). For all soils: a two-part soil one-part molecular grade water slurry was prepared. The mixture of soil and water was contained in a 50 ml VWR Falcon tube and was homogenized for 2 hours on a Fisher Vortex Genie at maximum speed. The slurries were then centrifuged at 2440 RPM in a Sorvall Super T21 benchtop centrifuge for 10 minutes at room temperature (28.8 °C ± 2 °C) to pellet any remaining soil debris. Subsequently, the slurry was aspirated from the Falcon tube, and serially diluted to prepare 10^{-6}, 10^{-7} and 10^{-8} dilutions. Aliquots of 50 ul and 100 ul of each slurry dilution were plated onto high population (>1,000) *C. elegans* culture plates (described in 4.1.2) to monitor for microbial pathogenesis and these slurries functioned as liquid suspensions of microorganisms present within the field soil tested. The studies were repeated three times using three replicates for each solution/dilution in order to locate as many nematophagous microbial candidates as possible. We have supplied a supplemental flow diagram of this experimental design with the re-submission of our manuscript (Supplementary Figure S1). The number of microbial isolates from each soil in preliminary screening can be viewed in Supplementary Figure S2.

By applying these slurries to cultures of *C. elegans in vitro*, we then examined any nematodes experiencing bacterial of fungal attacks. Nematodes thought to be infected by some microorganism were isolated into Luria Bertani agar (LBA) or potato dextrose agar (PDA) based on the presence of bacterial biofilm or fungal hyphae. After the initial screening process, we continued with a total of 16 potentially-nematophagous microbial isolates from three different soils (Supplemental Figure S2). Upon testing these microorganisms on cultures of *C. elegans* in isolation, our initial 16 candidates were reduced to 3; two fungal organisms and one bacterium. These microbes were from both the 'Sargent' and 'Mosca II' potato fields in the San Luis Valley, CO, and no candidate microbial isolates were used from the Blanca field.

Upon repeating the isolated pathogenicity assay on *C. elegans* with the 3 candidate microbes, we discovered that only one fungus was effectively (and rapidly) infecting *C. elegans* in vitro. This fungus was *M. globalpina*. The second candidate, a bacterium, did not show the same results upon repeating the experiment. The third candidate, a fungus, was observed to be antagonistic in the first trial; but on subsequent trials did not show bioactivity. Further, we conducted PCR on this isolate (using ITS1 universal fungal primer) and confirmed it to be *Rhizopus stolonifer,* a mold quick to colonize sugars and starches. *R. stolonifera* is not bioactive against nematodes.

Supplementary Figure S1: Method of identifying nematophagous microorganisms using *C. elegans* as a model for generalist pathogens of nematodes.

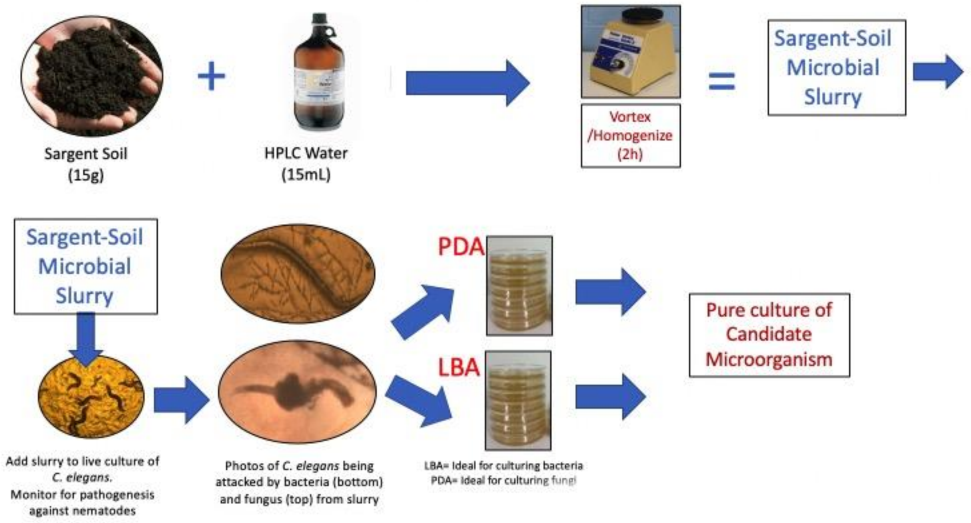

Supplementary Figure S2: Pie chart representing the initial number of infected *C. elegans* after each field-slurry application. Numbers denote number of worm infections from microorganisms within each soil.

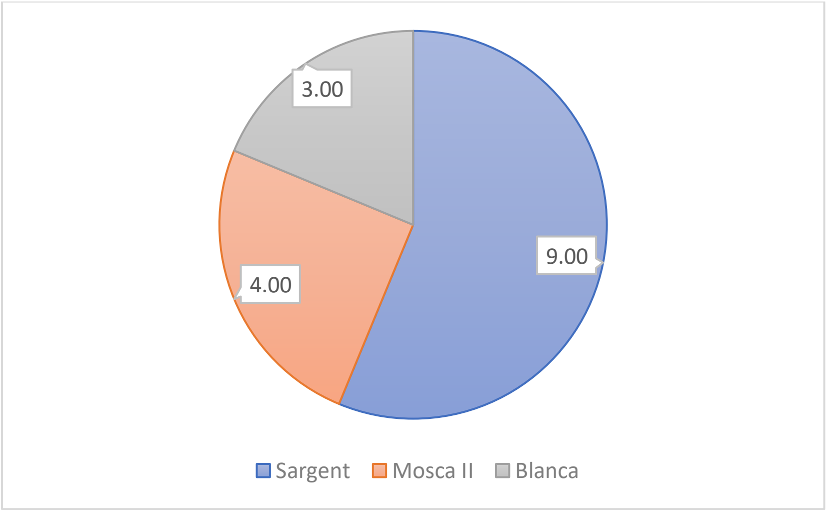

Supplementary Table S1: Results of the *in vitro* pathogenicity assessment of *M. globalpina* against both *C. elegans* and *M. chitwoodi.* Results are the average of 5 replicates and are displayed as mean ± standard deviation.

In vitro pathogenicity assessment of *M. globalpina* against two spp. of nematodes

Experiment (avg. of 5 reps)	Initial Eggs Added (eggs/ Petri dish)	Paralyzed Worm Count (20h)	Paralyzed Worm Count (42h)	Paralyzed Worm Count (72h)	% Paralyzed (72h)
In vitro Pathogenicity Assay: Caenorhabditis elegans plus Mortierella globalpina	11. 8 ± 3.03	8.6 ± 2.19	10.6 ± 1.82	11.8 ± 3.03	100%
In vitro Pathogenicity Assay: Meloidogyne chitwoodi plus Mortierella globalpina	Initial Eggs Added (eggs/ Petri dish)	Infected Worm Count (72h)	Living Worm Count (72h)	Eggs/ J2 Nematodes Colonized by fungus	% Infected by Fungus (72h)
	30.4 ± 8.26	6 ± 1.41	0.8 ± 0.84	23.8 ± 8.96	78%

Supplementary Table S2: Root-scan analysis of Rutgers var. tomato plants analyzed by WinRHIZO software. Results are the average of 15 replicates and are displayed as mean ± standard deviation.

WinRHIZO Root Scan Analysis (Avg. of 15 reps)

**Treatment**	Length (cm)	Avg. Diam (mm)	Root Volume (cm^3)	No. of Crossings	No. of Galls	No. of Forks	No. of Tips
**Control** *10,000 M. chitwoodi* eggs/plant	1,116.24± 716.1	1.1232 ± 1.2	10.183 ± 14.2	10,495.4 ± 9,416.8	114.4 ± 31.8	27,400.7 ± 13,993.8	2,762.4 ± 960

**Spores plus Eggs** *10,000 M. chitwoodi* eggs/plant plus $1x10^8$ *M. globalpina spores/ml*	1,201.07± 572.3	0.80212 ± 0.37	6.204 ± 4.9	10,065.8 ± 7,992.6	30.6 ± 7.6	24,886.1 ± 11,199.3	4,562.8 ± 2,597

Supplementary Image S1: Micrographs of experiment through bench-top microscope. **A**) Diagram of experimental design with *M. globalpina* and *M. chitwoodi*. **B**) *M. chitwoodi* eggs begin attacked by fungal hyphae of *M. globalpina.* **C**) Adult *M. chitwoodi* trapped in adhesive hyphae of *M. globalpina* (with hyphal sporulation). **D**) *M. chitwoodi* adult with fungal hyphae of *M. globalpina* penetrating nematode cuticle. **E**) Dead adult *M. chitwoodi* colonized by sporulating fungus. **F**) *M. chitwoodi* egg & larvae being attacked by *M. globalpina.*

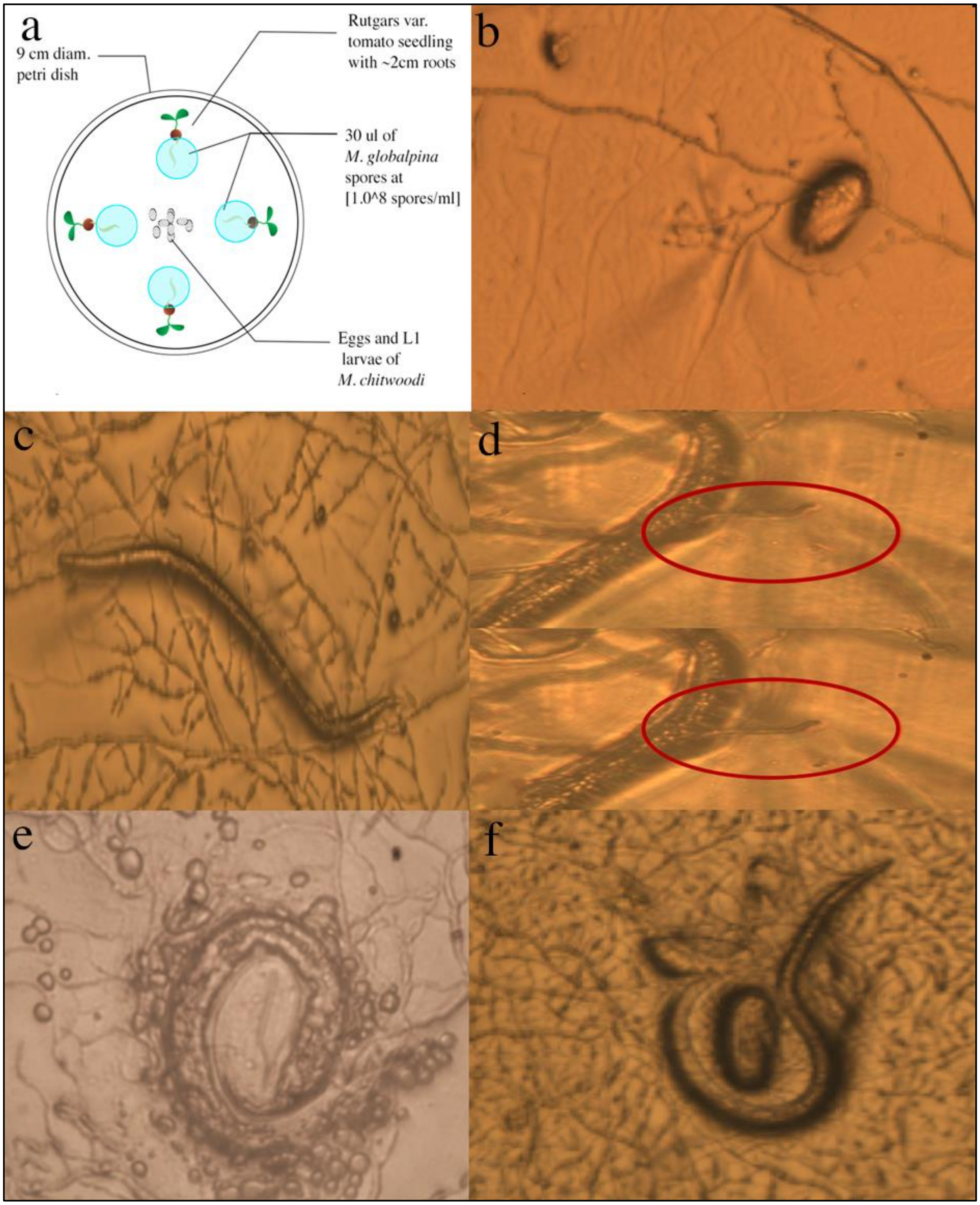
a
9 cm diam.
petri dish
Rutgars var.
tomato seedling
with ~2cm roots
30 ul of
M. globalpina
spores at
[1.0^8 spores/ml]
Eggs and L1
larvae of
M. chitwoodi
b
c
d
e
f
